# Differential Effects of Stroke Stage and Age on Sarcopenia in Stroke Patients: A Cross-Sectional Study

**DOI:** 10.3390/life16071073

**Published:** 2026-06-27

**Authors:** Guan-Bo Chen, I-Hsiu Liou, Shu-Fen Sun, Chien-Hui Li, Sheng-Hui Tuan

**Affiliations:** 1 Division of Gastroenterology, Department of Internal Medicine, Kaohsiung Armed Forces General Hospital, Kaohsiung 802, Taiwan; tanwein@gmail.com; 2 Division of Gastroenterology, Department of Internal Medicine, Tri-Service General Hospital, National Defense Medical Center, Taipei 114, Taiwan; 3 Department of Physical Medicine and Rehabilitation, Kaohsiung Veterans General Hospital, Kaohsiung 813, Taiwan; isliuvghks@gmail.com (I.-H.L.); tsaichiju@gmail.com (S.-F.S.); 4 Department of Physical Therapy, School of Medical and Health Science, Fooyin University, Kaohsiung 831, Taiwan; 5 Faculty of Medicine, College of Medicine, National Yang Ming Chiao Tung University, Yangming Campus, Taipei 112, Taiwan; 6 Department of Rehabilitation Medicine, Cishan Hospital, Ministry of Health and Welfare, Kaohsiung 842, Taiwan; leechpt100@gmail.com; 7 Department of Physical Medicine and Rehabilitation, School of Medicine, College of Medicine, Kaohsiung Medical University, Kaohsiung 807, Taiwan

**Keywords:** aging, functional performance, muscle mass, sarcopenia, stroke

## Abstract

Sarcopenia is highly prevalent among stroke patients and is associated with poor functional outcomes; however, differences across stroke stages and age groups remain unclear. This cross-sectional study enrolled 80 stroke patients from a regional teaching hospital in Taiwan, categorized into chronic (*n* = 40) and post-acute care (PAC) groups (*n* = 40), and further stratified into younger (40–64 years, *n* = 44) and older (≥65 years, *n* = 36) groups. Assessments included body composition, muscle strength, ultrasound-measured muscle thickness, gait speed, calf circumference, sarcopenia screening (SARC-F), nutritional status, and health-related quality of life. No significant differences were observed in muscle mass, muscle strength, or ultrasound-derived muscle thickness between the chronic and PAC groups. However, the PAC group demonstrated poorer functional outcomes and health-related quality of life, including lower gait speed (*p* = 0.018), and lower EQ-5D index and visual analogue scale scores (*p* = 0.006 and *p* = 0.002, respectively). In contrast, the chronic group showed a higher prevalence of sarcopenia (*p* < 0.001), a higher mean SARC-F scores (*p* = 0.004), a greater proportion of low appendicular skeletal muscle mass index (ASMMI, *p* = 0.025), and reduced calf circumference (*p* < 0.001). Age-stratified analysis revealed that older patients had lower muscle mass and structural parameters, including ASMMI (*p* < 0.001), fat-free mass (*p* < 0.001), quadriceps thickness (*p* < 0.001), and calf circumference (*p* = 0.002), along with a higher prevalence of sarcopenia (*p* < 0.001). These findings indicate that stroke stage is more closely associated with functional impairment, whereas aging predominantly affects muscle mass and sarcopenia severity.

## 1. Introduction

Sarcopenia is an age-related skeletal muscle disorder characterized by the progressive loss of muscle mass, strength, and physical performance, and is associated with increased risks of disability, hospitalization, and mortality [1]. The European Working Group on Sarcopenia in Older People (EWGSOP) first established a consensus definition in 2010, which substantially advanced research in this field [2]. Considering ethnic and body composition differences, the Asian Working Group for Sarcopenia (AWGS) updated its diagnostic criteria in 2019, defining sarcopenia as low muscle mass accompanied by low muscle strength or poor physical performance [3]. Based on these criteria, the prevalence of sarcopenia among Taiwanese adults aged ≥65 years is generally below 20% in community-dwelling populations but exceeds 50% in clinical or long-term care settings [4]. Individuals with sarcopenia also incur substantially higher healthcare costs than those with normal muscle mass [5]. The etiology of sarcopenia is multifactorial, involving aging, malnutrition, physical inactivity, and chronic diseases [1,3]. Hospitalization itself has been recognized as an iatrogenic contributor, with approximately 15% of patients developing new-onset sarcopenia during acute hospital stays [6]. Inadequate nutritional intake and reduced physical activity during illness may further accelerate muscle loss [7].

Stroke is a leading cause of death and long-term disability worldwide [8], with increasing survival rates and a trend toward younger onset [9]. Older stroke survivors often experience poorer outcomes, with more than 70% developing long-term disability in aging populations [9,10]. Therefore, a geriatric perspective is essential in stroke management. Stroke-related sarcopenia refers to the development or exacerbation of sarcopenia following stroke [11]. Potential mechanisms include muscle disuse due to hemiparesis, denervation, inflammation, and nutritional impairment [11,12]. Sarcopenia is highly prevalent among patients with stroke, ranging from 8.5% to 33.8% in acute settings [13,14] and up to 50% in post-acute care [15,16]. It is also associated with poorer functional recovery and increased adverse outcomes [17]. According to a meta-analysis, the pooled prevalence of sarcopenia among patients with stroke is estimated to be 42% (95% CI: 33–52%), with individual studies reporting rates ranging from 17% to 60% [18]. These findings highlight the substantial burden of sarcopenia in this population. Accordingly, early identification, prevention, and management of stroke-associated sarcopenia have emerged as critical priorities for contemporary healthcare providers.

Beyond muscle loss and physical impairment, sarcopenia has also been associated with reduced independence, poorer functional status, and diminished health-related quality of life [19]. In stroke survivors, the combined effects of neurological deficits and sarcopenia may further compromise perceived health status and rehabilitation outcomes [20]. However, few studies have simultaneously examined sarcopenia-related parameters and health-related quality of life across different stroke stages and age groups. Moreover, previous studies have primarily assessed sarcopenia using single measures, such as handgrip strength or calf circumference [21,22], whereas comprehensive multidimensional evaluations remain limited. In addition, age-related muscle decline begins around 40 years of age and may vary by sex [23]. Few studies have compared sarcopenia-related parameters between post-acute and chronic stroke populations. Therefore, this study aimed to (1) comprehensively evaluate sarcopenia-related variables using different clinical measurement tools in patients with post-acute and chronic stroke and (2) examine the influence of stroke stage and age group (40–65 vs. >65 years) on these parameters.

## 2. Materials and Methods

### 2.1. Study Design and Setting

This cross-sectional observational study was conducted at the outpatient clinics and post-acute care (PAC) wards of a regional teaching hospital affiliated with the Ministry of Health and Welfare, in southern Taiwan. Participants were consecutively recruited during outpatient visits or initial PAC admission assessments. Participants were recruited using a consecutive sampling strategy between March 2025 and October 2025, all sarcopenia classifications were based on the AWGS 2019 criteria. All patients who met the predefined inclusion and exclusion criteria during the study period were consecutively screened and invited to participate. Recruitment was not based on sex, stroke subtype, functional status, or sarcopenia severity, thereby minimizing potential selection bias. Clinical and demographic data were obtained through direct assessment and retrospective medical record review, conducted by an experienced physiatrist (S.H. Tuan) and a senior ward nurse manager (A.R. Fang). This study was approved by the Institutional Review Board of National Cheng Kung University Hospital (IRB No. A-ER-114-059). The study was registered at ClinicalTrials.gov (NCT07492849) and was conducted in accordance with the Strengthening the Reporting of Observational Studies in Epidemiology (STROBE) guidelines [24].

### 2.2. Participants

Participants were categorized into two groups: (1) patients with chronic stroke and (2) patients with post-acute stroke who were newly admitted to a post-acute care (PAC) program. Chronic stroke was defined as a duration of more than 1 year since the most recent stroke event. Post-acute stroke was defined as at least 14 days after the most recent stroke, with eligibility for transfer to a PAC program determined by a neurologist and confirmed admission to a PAC ward. In addition, subgroup analyses were performed by stratifying participants into younger (40 ≤ age < 65 years) and older (age ≥ 65 years) stroke groups.

Eligible participants met the following inclusion criteria: (1) age ≥40 years with a confirmed diagnosis of ischemic or hemorrhagic stroke, defined by specialist evaluation and corresponding International Classification of Diseases, 10th Revision (ICD-10) codes documented in the hospital medical record system; (2) sufficient cognitive function and physical capacity to complete sarcopenia-related assessments, including body composition, handgrip strength, gait speed, and the Mini Nutritional Assessment (MNA), as determined by the principal investigator; and (3) provision of informed consent. For participants in the chronic stroke group, informed consent was obtained directly from the patients. For those in the post-acute group, if cognitive impairment was present, consent was obtained from family members or legal representatives before assessment.

The exclusion criteria were as follows: (1) history of lower extremity neuromuscular or musculoskeletal disorders within the previous 6 months; (2) severe cardiopulmonary or neurological conditions requiring oxygen therapy; and (3) major infection requiring hospitalization and intravenous antibiotics or other invasive medical interventions, such as cardiac catheterization, within 1 month before enrollment. Sample size was estimated using G*Power (version 3.1) for a two-tailed test, assuming a large effect size (Cohen’s d = 0.8), with α = 0.05 and a statistical power of 0.80, yielding a minimum of 15 participants per group. The selection of a large effect size was based on previous rehabilitation and sarcopenia-related studies reporting moderate-to-large effect sizes for muscle strength, physical performance, gait-related outcomes, and functional recovery in older adults and stroke populations [25,26]. Considering an estimated incomplete assessment or dropout rate of approximately 30%, the required sample size was increased, and ultimately a minimum of 40 participants (20 per group) was required.

### 2.3. Outcome Measures

All participants underwent both objective and subjective assessments after enrollment. Given the cross-sectional design of the study, all measurements were performed at a single time point. For participants in the chronic stroke group, assessments were conducted at the time of outpatient enrollment, whereas for those in the PAC stroke group, assessments were performed three weeks after transfer from the acute care ward to the PAC ward. Objective assessments included sarcopenia-related parameters, namely body composition, the Strength, Assistance with walking, non-hemiparetic side handgrip strength (HGS), gait speed, quadriceps muscle strength, ultrasound-measured thickness of the quadriceps and gastrocnemius muscles, calf circumference, as well as nutritional status assessed using the MNA. Subjective assessment included Strength, Assistance with walking, Rise from a chair, Climb stairs, and Falls (SARC-F) questionnaire, and health-related quality of life (HRQoL),assessed using the EuroQol 5-Dimension 5-Level (EQ-5D-5L) questionnaire. All questionnaire-based and functional assessments, including SARC-F, MNA, EQ-5D, calf circumference, and gait speed, were performed by one trained physical therapist (C.H.Li) using standardized procedures. Ultrasound measurements were performed by an experienced physician (S.H.Tuan) to reduce measurement variability.

In this study, sarcopenia was defined according to the AWGS 2019 criteria [3] because no universally accepted stroke-specific diagnostic cut-off values for sarcopenia have yet been established. Although stroke-related sarcopenia is increasingly recognized, previous studies have noted that its assessment remains largely based on general sarcopenia frameworks, such as the EWGSOP or AWGS criteria [27,28]. Therefore, the AWGS 2019 criteria were applied to ensure consistency with Asian population-based standards and comparability with prior stroke-related sarcopenia studies. Low muscle mass was defined as an appendicular skeletal muscle mass index (ASMMI) <7.0 kg/m^2^ for men and <5.7 kg/m^2^ for women, as measured by bioelectrical impedance analysis. Low muscle strength was defined as handgrip strength <28 kg for men and <18 kg for women, and low physical performance was defined as gait speed <1.0 m/s. Participants with low muscle mass plus either low muscle strength or low physical performance were classified as having sarcopenia, whereas those fulfilling all three criteria were classified as having severe sarcopenia. Possible sarcopenia was defined as low muscle strength with or without reduced physical performance. The SARC-F questionnaire was used only as a screening tool for sarcopenia risk and was not used for diagnostic classification.

#### 2.3.1. Body Composition

Body composition was assessed using a multi-frequency bioelectrical impedance analysis (BIA) device, which estimates impedance and derives body fat, fat-free mass, total body weight, intracellular water, and extracellular water [29]. Compared with conventional single-frequency BIA, multi-frequency BIA provides a more accurate assessment of body composition [30]. BIA was selected because it is simple, non-invasive, clinically feasible, and widely used for estimating appendicular skeletal muscle mass, although DXA remains the reference standard for body composition assessment [31]. Body mass index (BMI) was calculated as body weight (kg) divided by height squared (m^2^). Fat-free mass index (FFMI) was calculated as fat-free mass (kg) divided by height squared (m^2^). Appendicular skeletal muscle mass index (ASMMI) was calculated as appendicular skeletal muscle mass (kg) divided by height squared (m^2^).

#### 2.3.2. SARC-F Questionnaire

SARC-F questionnaire is a validated screening tool for sarcopenia. It consists of five self-reported items, with a total score ranging from 0 to 10, where higher scores indicate a greater risk of sarcopenia. A positive SARC-F screening result was defined as a score ≥4. The SARC-F has demonstrated good validity and reliability in predicting adverse health outcomes, including hospitalization, functional decline, and mortality, and is widely used for sarcopenia screening in clinical and community settings [32].

#### 2.3.3. Non-Paretic Side Handgrip Strength

Handgrip strength was measured using a JAMAR dynamometer under standardized positioning from the non-paretic side. Participants were seated with the shoulder adducted, elbow flexed at 90°, forearm in a neutral position, and wrist slightly extended. Three trials were performed with a 1-min rest between trials, and the highest value was recorded for analysis [33].

#### 2.3.4. Quadriceps Muscle Strength

Quadriceps muscle strength was assessed as maximal voluntary isometric contraction (MVIC) using a handheld dynamometer (MicroFET3; Hoggan Health Industries, UT, USA) under standardized conditions. The mean value of two trials was recorded. Participants were instructed to perform maximal knee extension against resistance [34]. Each measurement was repeated after a 1-min rest period, and the average value was used for analysis.

#### 2.3.5. Sonographic Muscle Thickness of Non-Paretic Lower Extremity

Muscle thickness of the non-paretic lower extremity was assessed using portable ultrasonography (LOGIQ e; General Electric Company, USA) with a 12-MHz linear probe. All measurements were performed by the same experienced examiner (S.H. Tuan) under standardized positioning to ensure measurement consistency. Quadriceps thickness was defined as the sum of the rectus femoris and vastus intermedius measured on transverse scans [35]. Gastrocnemius assessment included both fat thickness (distance from the superficial fascia to the skin) and muscle thickness (distance from the superficial to deep fascia), measured in the axial plane [36]. Previous studies have demonstrated that quadriceps and gastrocnemius muscle thickness are positively correlated with lower extremity muscle strength, and that individuals with sarcopenia may exhibit early atrophy of these muscle groups [37,38].

#### 2.3.6. Gait Speed and Calf Circumference

Gait speed was assessed using a 6-m walk test. This distance was selected because it is routinely used in our rehabilitation department and is feasible for stroke patients with mobility limitations. The 6-m distance provides sufficient walking length to capture usual walking speed while minimizing excessive fatigue and space requirements in clinical settings [39]. Participants were instructed to walk at their usual comfortable speed, and the mean value of two trials was used for analysis. Calf circumference was measured bilaterally, including both the paretic and non-paretic limbs, under standardized conditions. To minimize the influence of edema, measurements were performed in the morning. Each side was measured twice, and the highest value was recorded [22]. Previous studies have suggested that calf circumference thresholds of <31–34 cm for men and <30–33 cm for women are indicative of sarcopenia in stroke populations [22].

#### 2.3.7. Nutritional and Health Related Quality of Life Assessment

Nutritional status was evaluated using the MNA, an 18-item tool comprising screening and assessment components, with a total score ranging from 0 to 30. Scores ≥24 indicate normal nutritional status, 17–23.5 indicate a risk of malnutrition, and <17 indicate malnutrition [40]. HRQoL was assessed using the EQ-5D-5L questionnaire. The EQ-5D-5L index score was calculated using the value set developed by Luo et al. based on a regression model, with scores ranging from −0.391 to 1.000, where 1.000 represents full health, 0 corresponds to death, and negative values indicate health states worse than death [41]. In addition, a visual analogue scale (VAS) ranging from 0 (worst health) to 100 (best health) was recorded [41].

### 2.4. Statistical Analysis

Statistical analyses were performed using SPSS software (version 19.0; IBM Corp., Armonk, NY, USA). All tests were two-tailed, and a *p*-value < 0.05 was considered statistically significant. Continuous variables are presented as mean ± standard deviation (SD), whereas categorical variables are presented as counts and percentages.

Participants were primarily stratified by stroke stage (chronic vs. PAC) and further analyzed using age-based subgroup classification (younger: 40–64 years; older: ≥65 years). Between-group comparisons of continuous variables (e.g., body composition, muscle strength, and functional parameters) were performed using Welch’s *t*-test, which does not assume equal variances and provides more robust estimates in the presence of heterogeneity between groups. Categorical variables, including sarcopenia-related classifications (e.g., ASMMI, grip strength, gait speed, SARC-F, calf circumference, and overall sarcopenia status), were analyzed using the chi-square test.

## 3. Results

### 3.1. Baseline Characteristics of the Study Population

A total of 80 participants were enrolled after applying the inclusion and exclusion criteria. Participants were categorized according to stroke stage into a chronic stroke group (*n* = 40) and a post-acute care (PAC) stroke group (*n* = 40). The mean duration since stroke onset was 1886.70 ± 1238.77 days in the chronic stroke group and 49.61 ± 39.20 days in the PAC group (*p* < 0.001). The chronic stroke group had a median stroke duration of 1541 days (IQR: 834–3020 days; range: 409–4661 days), whereas the PAC group had a median stroke duration of 29 days (IQR: 21–76 days; range: 12–138 days), indicating marked differences in stroke chronicity between the two groups. In the PAC group, 32 patients had ischemic stroke and 8 had hemorrhagic stroke, whereas in the chronic stroke group, 30 patients had ischemic stroke and 10 had hemorrhagic stroke (*p* = 0.789).

Additionally, subgroup analyses were performed by stratifying participants into younger (40–64 years, *n* = 44) and older (≥65 years, *n* = 36) groups.

Table 1 presents the baseline characteristics of the study population stratified by stroke stage and age group. No significant differences were observed between the chronic and PAC stroke groups in terms of demographic characteristics, including age, sex distribution, height, body weight, and body mass index, except for the duration since stroke onset, which was significantly longer in the chronic stroke group (*p* < 0.001). Similarly, when stratified by age, the younger and older groups showed significant differences in age, height, and body weight (all *p* < 0.001), whereas no significant differences were observed in sex distribution, duration since stroke, or body mass index.

### 3.2. Comparisons of Body Composition and Sarcopenia-Related Parameters Between Groups

Table 2 presents the comparisons of body composition and sarcopenia-related parameters stratified by stroke stage and age group. When stratified by stroke stage, most body composition parameters, including visceral fat, subcutaneous fat distribution, ASMM, ASMMI, fat mass, and fat-free mass, showed no significant differences between the chronic and PAC stroke groups. No significant differences were observed in muscle strength or ultrasound-derived muscle thickness between the two groups. However, significant differences were observed in several functional and clinical indicators. Compared with the PAC group, the chronic stroke group had significantly higher SARC-F scores (*p* = 0.004), indicating greater self-reported sarcopenia-related functional difficulty. Conversely, the PAC group showed significantly lower EQ-5D index scores (*p* = 0.006), EQ-5D VAS scores (*p* = 0.002), and gait speed (*p* = 0.018), indicating poorer health-related quality of life and walking performance during the early recovery phase.

In contrast, age-stratified analysis revealed significant differences in several muscle-related parameters. The younger group exhibited significantly higher ASMM (*p* < 0.001) and fat-free mass (*p* < 0.001) compared with the older group, indicating greater muscle mass. Ultrasound measurements also showed significantly greater quadriceps muscle thickness in the younger group (*p* < 0.001). In addition, calf circumference was significantly larger in the younger group (*p* = 0.002), further supporting better muscle status. Other variables, including ASMMI, FFMI, HGS, and gait speed, showed trends toward better performance in the younger group, although these differences did not reach statistical significance.

### 3.3. Comparisons of Sarcopenia-Related Categorical Indicators Between Groups

Table 3 presents the comparisons of sarcopenia-related categorical indicators stratified by stroke stage and age group. When stratified by stroke stage, a significant difference was observed in ASMMI classification, with a higher proportion of low ASMMI in the chronic stroke group compared with the PAC group (*p* = 0.025). However, no significant differences were found in grip strength or gait speed classifications between the two groups. Based on the AWGS 2019 diagnostic criteria, the distribution of sarcopenia status differed significantly between groups (*p* < 0.001). The chronic stroke group demonstrated a higher proportion of sarcopenia and severe sarcopenia, whereas the PAC group showed a predominance of possible sarcopenia. Regarding screening indicators, no significant difference was observed in SARC-F classification between groups (*p* = 0.369). In contrast, calf circumference classification differed significantly, with a higher proportion of low calf circumference in the chronic stroke group compared with the PAC group (*p* < 0.001), indicating greater muscle mass reduction in chronic stroke patients.

In the age-stratified analysis, significant differences were observed in grip strength classification (*p* = 0.011), with a higher proportion of low grip strength in the older group. Although ASMMI and gait speed classifications did not reach statistical significance, trends toward poorer performance were observed in the older group. Furthermore, the distribution of sarcopenia status differed significantly between age groups (*p* < 0.001), with the older group demonstrating a markedly higher proportion of sarcopenia and severe sarcopenia. Similarly, calf circumference classification showed a significant difference (*p* < 0.001), with a greater proportion of low values in the older group. No significant difference was observed in SARC-F classification (*p* = 0.072).

## 4. Discussion

In this cross-sectional study, stroke stage and age showed distinct associations with sarcopenia-related outcomes. Stroke stage was more closely related to functional performance and health-related quality of life, whereas age had a greater impact on muscle mass and structural parameters. Although chronic and PAC stroke patients showed comparable body composition and muscle morphology, PAC patients had poorer gait performance and lower EQ-5D scores, while older patients had lower muscle mass, quadriceps thickness, calf circumference, and higher sarcopenia severity. These findings suggest that stroke stage mainly reflects functional impairment, whereas aging predominantly contributes to muscle loss and sarcopenia severity.

In the present study, comparisons between chronic and post-acute stroke groups revealed a dissociation between objective muscle parameters and functional outcomes. Although muscle mass, strength, and ultrasound-derived thickness were comparable, the chronic stroke group exhibited higher SARC-F scores, a greater proportion of low ASMMI and reduced calf circumference, and a higher prevalence of sarcopenia and severe sarcopenia. These findings suggest that sarcopenia progression after stroke may not be fully captured by isolated quantitative muscle measurements, but rather reflects a cumulative decline influenced by long-term disuse, neuromuscular impairment, and metabolic alterations [42,43]. Chronic stroke patients are particularly vulnerable to disuse atrophy due to prolonged inactivity and asymmetrical limb loading, especially in the lower extremities, which play a critical role in mobility [44,45]. Additionally, chronic systemic inflammation and comorbid conditions such as diabetes and cardiovascular disease may further exacerbate muscle catabolism and functional decline [46]. In contrast, although patients in the PAC stage demonstrated relatively preserved sarcopenia profiles, they exhibited significantly poorer functional performance and HRQoL, including lower gait speed, EQ-5D index, and VAS scores. This pattern likely reflects the early post-stroke recovery phase, during which patients experience acute functional limitations, psychological distress, and reduced independence despite not yet developing substantial muscle loss [47].

Age-stratified analyses showed that aging has a greater impact on muscle mass and structure. Older stroke patients had lower ASMM, fat-free mass, quadriceps thickness, and calf circumference, along with a higher prevalence of sarcopenia and severe sarcopenia. These findings are consistent with previous studies indicating that aging and stroke have synergistic effects on skeletal muscle deterioration [48]. The mechanisms underlying this phenomenon are multifactorial. Age-related loss of muscle mass and strength is driven by reductions in muscle fiber number and size, impaired mitochondrial function, and decreased anabolic responsiveness. These changes are further aggravated by stroke-related inactivity and neuromuscular dysfunction [49]. In addition, chronic low-grade inflammation hormonal changes, and impaired neuromuscular transmission contribute to accelerated muscle degradation in older individuals [50]. Importantly, calf circumference demonstrated strong discriminative ability for identifying age-related sarcopenia risk in this study, with a significantly higher proportion of low values in the older group. This supports previous evidence suggesting that calf circumference is a practical and sensitive screening tool for sarcopenia in clinical settings, particularly in older populations [51]. However, calf circumference may be influenced by age, sex, body composition, and population-specific characteristics, and therefore should not be interpreted as a definitive diagnostic measure in stroke survivors. Compared with a single anthropometric cutoff, the AWGS criteria provide a more comprehensive assessment by integrating muscle mass, muscle strength, and physical performance. Future studies should further evaluate the diagnostic performance of calf circumference and determine whether stroke-specific screening thresholds are needed. In addition, although grip strength and gait speed did not reach statistical significance, both showed consistent trends toward poorer performance in older patients, further supporting the impact of aging on functional capacity.

One possible explanation for the observed dissociation between sarcopenia prevalence and functional outcomes is that structural and functional consequences of stroke may evolve along different timelines. Stroke-related sarcopenia is increasingly recognized as a progressive condition driven by prolonged physical inactivity, impaired neural activation, chronic low-grade inflammation, and adverse muscle remodeling following stroke [11,52]. These processes may gradually accumulate over years after stroke, resulting in reductions in muscle mass and muscle quality among chronic stroke survivors. However, structural deterioration does not necessarily translate immediately into measurable functional decline. Previous studies have suggested that chronic stroke survivors may develop compensatory movement strategies, adapt to long-standing physical limitations, and benefit from accumulated rehabilitation experience, thereby partially preserving daily function despite progressive muscle loss [53]. Consequently, sarcopenia-related structural changes may accumulate silently for prolonged periods before their functional consequences become fully apparent. This concept may help explain why the chronic stroke group in the present study exhibited a higher prevalence of sarcopenia despite relatively comparable muscle-related measurements and functional performance compared with the PAC group.

The findings observed in the PAC group may also have important implications for rehabilitation timing. Despite relatively preserved muscle-related parameters, PAC patients demonstrated poorer gait performance and lower health-related quality of life, suggesting that functional limitations during the early recovery phase may be driven more by neurological impairment, reduced confidence, and incomplete adaptation than by substantial muscle loss [54,55]. This period may therefore represent a critical window of opportunity for intervention because neurological recovery and neuroplasticity are particularly active during the early post-stroke stage [55]. Intensive rehabilitation focusing on functional retraining, mobility enhancement, task-specific practice, and psychological support may therefore yield substantial improvements in recovery [56]. Early intervention during this stage may also help prevent subsequent chronic disuse, muscle atrophy, and stroke-related sarcopenia [11].

Several limitations should be acknowledged. First, although the sample size met the minimum requirement based on power analysis, it remained modest and may have limited the detection of subtle differences, particularly in subgroup analyses. Sex-related differences in muscle mass, strength, hormonal regulation, and sarcopenia progression have been reported in both aging and stroke populations; however, the sample size was insufficient to support reliable sex-stratified analyses. Second, the cross-sectional design precludes causal inference, and differences between PAC and chronic stroke groups may partly reflect variations in recovery trajectories and rehabilitation exposure. Third, heterogeneity existed within the PAC group because the duration since stroke onset varied among participants. Fourth, participants were recruited from a single regional teaching hospital in southern Taiwan. The chronic group primarily consisted of stable long-term survivors, whereas the PAC group was enrolled from a structured post-acute rehabilitation program; therefore, the findings may not be fully generalizable to community-dwelling stroke survivors or populations with limited rehabilitation access. Fifth, some assessments, including SARC-F and EQ-5D, relied on self-reported measures and may be subject to reporting bias. Finally, multivariable adjustment was not performed, and residual confounding cannot be excluded. In addition, validated stroke-specific sarcopenia cut-off values remain unavailable; therefore, AWGS criteria were used. Future longitudinal, multicenter studies with larger samples are warranted to examine the relationships among stroke severity, National Institutes of Health Stroke Scale (NIHSS), sarcopenia progression, and functional recovery. Further sex-specific analyses and validation of stroke-specific diagnostic criteria may improve risk stratification and support individualized rehabilitation strategies for stroke survivors.

## 5. Conclusions

In conclusion, stroke stage and age exert distinct yet complementary effects on sarcopenia in stroke populations. Stroke stage is primarily associated with functional impairment and reduced health-related quality of life, whereas aging predominantly contributes to muscle loss, structural decline, and increased sarcopenia severity. Chronic stroke patients showed a higher burden of sarcopenia despite comparable objective muscle parameters, while post-acute patients exhibited greater functional limitations during early recovery. Older patients were characterized by reduced muscle mass and a higher prevalence of sarcopenia, reflecting the combined effects of aging and stroke. These findings highlight the importance of comprehensive assessment incorporating both functional and morphological parameters. Clinically, individualized rehabilitation strategies tailored to stroke stage and age are essential to optimize sarcopenia management.

## Figures and Tables

**Table 1:** Baseline characteristics of participants stratified by stroke stage and age group.

Variables	Chronic Stroke (*n* = 40)	Post-Acute Stroke (*n* = 40)	*p*-Value	Younger Stroke (*n* = 44)	Older Stroke (*n* = 36)	*p*-Value
Sex (M:F)	20:20	28:12	0.206	32:12	18:18	0.140
Days since stroke	1886.70 ± 1238.77 (IQR: 833.8–3020.3 Range: 409–4661)	49.61 ± 39.20 (IQR: 20.5–76.0 Range: 12–138)	<0.001 *	1183.86 ± 1465.82	786.38 ± 985.57	0.152
Age (years)	61.65 ± 11.48	62.22 ± 11.57	0.826	53.64 ± 6.28	73.31 ± 4.98	<0.001 *
Height (cm)	161.50 ± 10.93	164.89 ± 6.13	0.092	166.34 ± 9.11	156.80 ± 7.25	<0.001 *
Weight (kg)	65.98 ± 14.83	67.30 ± 14.59	0.689	71.19 ± 15.20	58.83 ± 9.57	<0.001 *
BMI (kg/m^2^)	25.07 ± 3.83	24.63 ± 4.41	0.635	25.52 ± 4.05	23.96 ± 3.81	0.081

Chronic stroke was defined as >1 year since the most recent stroke event. Post-acute stroke was defined as ≥14 days since the most recent stroke with confirmed admission to a post-acute care program. Younger stroke was defined as age 40–64 years, and older stroke as age ≥65 years. * *p* < 0.05.

**Table 2:** Comparisons of body composition and sarcopenia-related parameters stratified by stroke stage and age group.

Variables	Chronic ^a^ (*n* = 40)	PAC ^a^ (*n* = 40)	*p*-Value	Younger ^b^ (*n* = 44)	Older ^b^ (*n* = 36)	*p*- Value
Visceral fat	10.35 ± 3.89	9.67 ± 2.24	0.328	9.75 ± 3.59	10.70 ± 3.09	0.412
SC fat, left UE (kg)	1.32 ± 0.57	1.06 ± 0.55	0.121	1.25 ± 0.59	1.19 ± 0.56	0.648
SC fat, right UE (kg)	1.30 ± 0.59	1.03 ± 0.57	0.118	1.23 ± 0.60	1.16 ± 0.58	0.602
SC fat, left LE (kg)	2.83 ± 0.92	2.42 ± 0.89	0.091	2.77 ± 0.93	2.55 ± 0.92	0.428
SC fat, right LE (kg)	2.81 ± 0.95	2.44 ± 0.92	0.118	2.78 ± 0.97	2.52 ± 0.91	0.301
SC fat, trunk (kg)	10.18 ± 3.85	8.80 ± 4.31	0.168	10.35 ± 4.03	8.66 ± 3.89	0.114
ASMM (kg)	18.54 ± 5.87	20.53 ± 4.45	0.142	20.98 ± 5.33	16.42 ± 4.47	<0.001 *
ASMMI (kg/m^2^)	6.93 ± 1.33	7.51 ± 1.40	0.182	7.46 ± 1.24	6.60 ± 1.41	0.061
Fat mass (kg)	19.81 ± 7.48	17.11 ± 7.66	0.139	19.75 ± 7.72	17.47 ± 7.32	0.209
Fat mass index	7.67 ± 3.01	6.26 ± 2.73	0.082	7.12 ± 2.72	7.27 ± 3.42	0.873
Fat-free mass (kg)	46.17 ± 11.67	50.19 ± 9.80	0.118	51.44 ± 10.76	41.36 ± 8.70	<0.001 *
FFMI (kg/m^2^)	17.42 ± 2.42	18.37 ± 2.95	0.143	18.40 ± 2.47	16.70 ± 2.56	0.052
SARC-F score	4.92 ± 2.68	2.79 ± 2.30	0.004 *	3.00 ± 2.57	4.46 ± 2.57	0.089
EQ-5D index	0.78 ± 0.17	0.65 ± 0.12	0.006 *	0.77 ± 0.16	0.68 ± 0.16	0.082
EQ-5D VAS	78.95 ± 11.00	69.17 ± 11.65	0.002 *	74.12 ± 10.04	76.43 ± 14.47	0.412
Grip strength (kg)	26.05 ± 10.87	27.13 ± 12.46	0.701	29.20 ± 10.06	22.30 ± 12.31	0.054
Quadriceps MVIC (kg)	3.42 ± 1.01	3.73 ± 0.77	0.182	3.53 ± 0.83	3.53 ± 1.12	0.998
Quadriceps thickness (cm)	2.80 ± 0.92	3.07 ± 0.88	0.231	3.39 ± 0.82	2.35 ± 0.60	<0.001 *
Gastrocnemius thickness (cm)	1.53 ± 1.26	1.54 ± 0.21	0.964	1.65 ± 1.47	1.39 ± 0.50	0.318
Calf circumference (cm)	31.50 ± 3.99	33.33 ± 4.87	0.106	34.25 ± 4.58	30.50 ± 3.80	0.002 *
MNA score	27.08 ± 1.86	27.20 ± 1.56	0.768	27.29 ± 1.60	27.50 ± 1.65	0.532
Gait speed (m/s)	0.65 ± 0.39	0.44 ± 0.36	0.018 *	0.67 ± 0.38	0.49 ± 0.38	0.066

Data are presented as mean ± standard deviation. ASMM, appendicular skeletal muscle mass; ASMMI, appendicular skeletal muscle mass index; FFMI, fat-free mass index; MVIC, maximal voluntary isometric contraction; SC fat, subcutaneous fat; UE, upper extremity; LE, lower extremity; MNA, Mini Nutritional Assessment; EQ-5D, EuroQol 5-Dimension questionnaire; VAS, visual analogue scale; SARC-F, Strength, Assistance with walking, Rise from a chair, Climb stairs, and Falls questionnaire. ^a^ Chronic stroke was defined as >1 year since the most recent stroke event. Post-acute stroke (PAC) was defined as ≥14 days since the most recent stroke with confirmed admission to a post-acute care program. ^b^ Younger stroke was defined as age 40–64 years, and older stroke as age ≥65 years. * *p* < 0.05.

**Table 3:** Comparisons of sarcopenia-related categorical indicators stratified by stroke stage and age group.

Variables	Chronic ^a^ (*n* = 40)		PAC ^a^ (*n* = 40)		*p*-Value	Younger ^b^ (*n* = 44)		Older ^b^ (*n* = 36)		*p*- Value
	Low	Normal	Low	Normal		Low	Normal	Low	Normal	
ASMMI	12	28	4	36	0.025 *	8	36	14	22	0.057
Grip strength	20	20	12	28	0.068	16	28	24	12	0.011 *
Gait speed	36	4	36	4	1.000	26	18	28	8	0.088
SARC-F	24	16	20	20	0.369	16	28	22	14	0.072
Calf circumference	22	18	4	36	<0.001 *	20	24	30	6	<0.001 *
**Severity of Sarcopenia**										
**Category**	**Chronic (*n* = 40)**		**PAC (*n* = 40)**		***p*** **-value**	**Younger (*n* = 44)**		**Older (*n* = 36)**		***p*** **-value**
Severe sarcopenia	6		4		<0.001 *	2		10		<0.001 *
Sarcopenia	18		4			6		16		
Possible sarcopenia	12		28			18		2		
Non-sarcopenia	6		4			18		8		

Data are presented as number of participants. ASMMI, appendicular skeletal muscle mass index; SARC-F, Strength, Assistance with walking, Rise from a chair, Climb stairs, and Falls questionnaire. Cut-off values were defined according to the AWGS 2019 criteria. Low ASMMI was defined as <7.0 kg/m^2^ for men and <5.7 kg/m^2^ for women. Low grip strength was defined as <28 kg for men and <18 kg for women. Low gait speed was defined as <1.0 m/s. A positive SARC-F screening result was defined as a score ≥4. Low calf circumference was defined as <34 cm for men and <33 cm for women. Sarcopenia and severe sarcopenia were classified according to the AWGS 2019 criteria. Sarcopenia was defined as low muscle mass plus either low muscle strength or low physical performance, whereas severe sarcopenia was defined as the presence of low muscle mass, low muscle strength, and low physical performance. Possible sarcopenia was defined as low muscle strength with or without reduced physical performance. ^a^ Chronic stroke was defined as >1 year since the most recent stroke event. Post-acute stroke (PAC) was defined as ≥14 days since the most recent stroke with confirmed admission to a post-acute care program. ^b^ Younger stroke was defined as age 40–64 years, and older stroke as age ≥65 years. * *p* < 0.05.

## Data Availability

All data generated or analyzed during this study are included in this published article. Additional datasets are available from the corresponding author upon reasonable request at pj73010@hotmail.com.

## Abbreviations

The following abbreviations are used in this manuscript:

EWGSOP	European Working Group on Sarcopenia in Older People
AWGS	Asian Working Group for Sarcopenia
PAC	Post-acute care
STROBE	Strengthening the Reporting of Observational Studies in Epidemiology
ICD-10	International Classification of Diseases, 10th Revision
MNA	Mini Nutritional Assessment
SARC-F	Strength, Assistance with walking, Rise from a chair, Climb stairs, and Falls
HRQoL	Health-related quality of life
EQ-5D-5L	EuroQol 5-Dimension 5-Level
BIA	Bioelectrical impedance analysis
BMI	Body mass index
FFMI	Fat-free mass index
ASMMI	Appendicular skeletal muscle mass index
HGS	Handgrip strength
MVIC	Maximal voluntary isometric contraction
VAS	Visual analogue scale
SD	Standard deviation
